# CT sinus imaging utilization in Norway: trends over time and variation by region, age, and sex

**DOI:** 10.1007/s43999-026-00094-4

**Published:** 2026-09-02

**Authors:** Jan Porthun, Stine Andenæs, Ingrid Øfsti Brandsæter, Bjørn Hofmann, Elin Kjelle

**Affiliations:** 1 Norwegian University of Science and Technology, Campus Gjøvik, Gjøvik, Norway; 2 Radiography and Lighting Design, Department of Optometry, Faculty of Health and Social Sciences, University of South-Eastern Norway, Drammen, Norway; 3 Centre of Medical Ethics, University of Oslo, Oslo, Norway

**Keywords:** Low-value, CT sinus, Choosing Wisely campaign, Geographical variation, Radiology

## Abstract

**Background:**

Computed tomography (CT) of the sinuses is a standard component of radiological diagnostics. CT of the sinuses are often regarded as low-value care owing to their minimal impact on disease management. The aim of this study is to describe geographical and temporal variations in outpatient CT sinus utilisation in Norway from 2013 to 2023.

**Methods:**

Administrative data on outpatient CT sinus performed in Norway between 2013 and 2023 were analysed. Descriptive statistics were performed on both unadjusted and adjusted data. Measures of geographical variation applied were: the coefficient of variation (CV), the extremal quotient (EQ), and the systematic component of variation (SCV). Sex-specific differences were formally examined by comparing female and male rates using Mantel–Haenszel standardised statistical measures.

**Results:**

Between 2013 and 2023, the sex- and age-adjusted rates of CT sinus per 10,000 individuals in Norway decreased from 52 to 46. The EQ increased from 2.42 to 3.86, the CV from 26% to 31% and SCV values from 6.10 to 8.76, indicating a high level of geographical variation. The ratio of female to male for CT sinus is 1.42 (SD ± 0.11).

**Conclusions:**

The decline in CT sinus is partially linked to the coronavirus pandemic and is likely attributable to the ‘Choosing Wisely’ campaign. Geographical variability, evident from 2013 to 2015 and becoming more pronounced through 2023, indicates differing CT sinus routines across hospital catchment areas in Norway. It is possible that a substantial proportion of examinations conducted on women may constitute low-value care.

**Graphical Abstract:**

**Figure:**
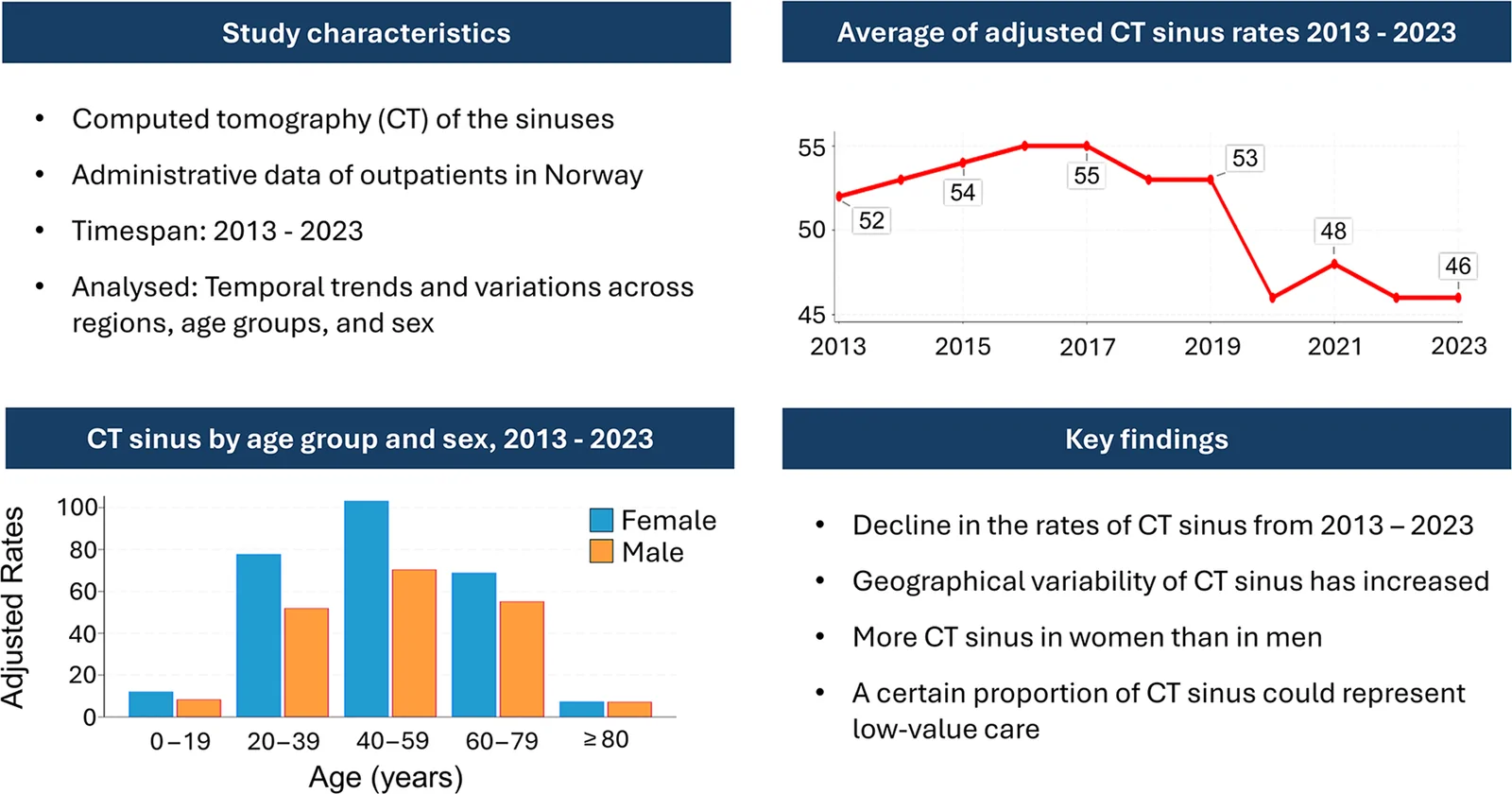

**Supplementary Information:**

The online version contains supplementary material available at https://doi.org/10.1007/s43999-026-00094-4.

## Background

Diagnostic imaging plays an essential role in the diagnosis, surveillance, and treatment of patients [1]. International research indicates that the use of healthcare services and diagnostic imaging technologies is increasing [2–4]. At the same time, unnecessary procedures and procedures of limited value to the patient are an increasing concern [5–9]. A low-value examination is one that has little or no effect on the management of the disease or positive health outcome [10]. The definition of low-value imaging, as provided by Hofmann, is as follows: ‘Low value imaging is imaging that does not contribute to reducing the overall pain, dysfunction or suffering of the person that is examined (compared to alternative actions)’ [11].

Research suggests that up to 50% of imaging procedures are unnecessary or overutilized [12, 13]. Furthermore, such examinations utilize healthcare resources, including imaging equipment and the time of radiographers and radiologists. Furthermore, such examinations seize healthcare resources which could have benefited others, including imaging equipment and the time of radiographers and radiologists. They generate long waiting times and substantial opportunity costs [14]. From a societal perspective, escalating costs threaten the sustainability of a viable healthcare system. Furthermore, exposure to ionizing radiation and contrast media elevates the risk to patients. Thus, to contribute to a sustainable health service, practitioners should only consider procedures that are highly valuable to patients.

The issue of how diagnostic imaging is employed and distributed across the population is gaining prominence. The Organization for Economic Co-operation and Development (OECD) widely acknowledges geographical variations as a challenge in attaining equitable access, quality, and efficiency in healthcare services [15]. Variations in imaging utilization across regions may occasionally indicate low-value imaging, as these patterns often reflect local practices. Previous research has demonstrated notable regional disparities in diagnostic imaging, not only within Norway but also in other countries [16–22]. These unwarranted geographical and temporal variations underscore the necessity for enhanced uniformity and suitability in imaging practices. Unjustified discrepancies in imaging utilisation across different regions are an indication of both overuse and underuse. Thereby it unfavourably affects the quality, safety, and efficiency of healthcare services.

Computed tomography (CT) can be employed for a wide range of examinations, including interventions, fracture monitoring, and oncological imaging. A CT scan provides important benefits to the patient when the resulting images facilitate accurate diagnosis and influence subsequent treatment decisions. However, it involves exposure to ionizing radiation and, in some cases, the administration of contrast media or other pharmaceuticals. Consequently, there are inherent risks for the individual patient [15, 23–25]. Data have demonstrated a steady increase over time in the utilization of CT [15, 26–29].

**Fig. 1:**
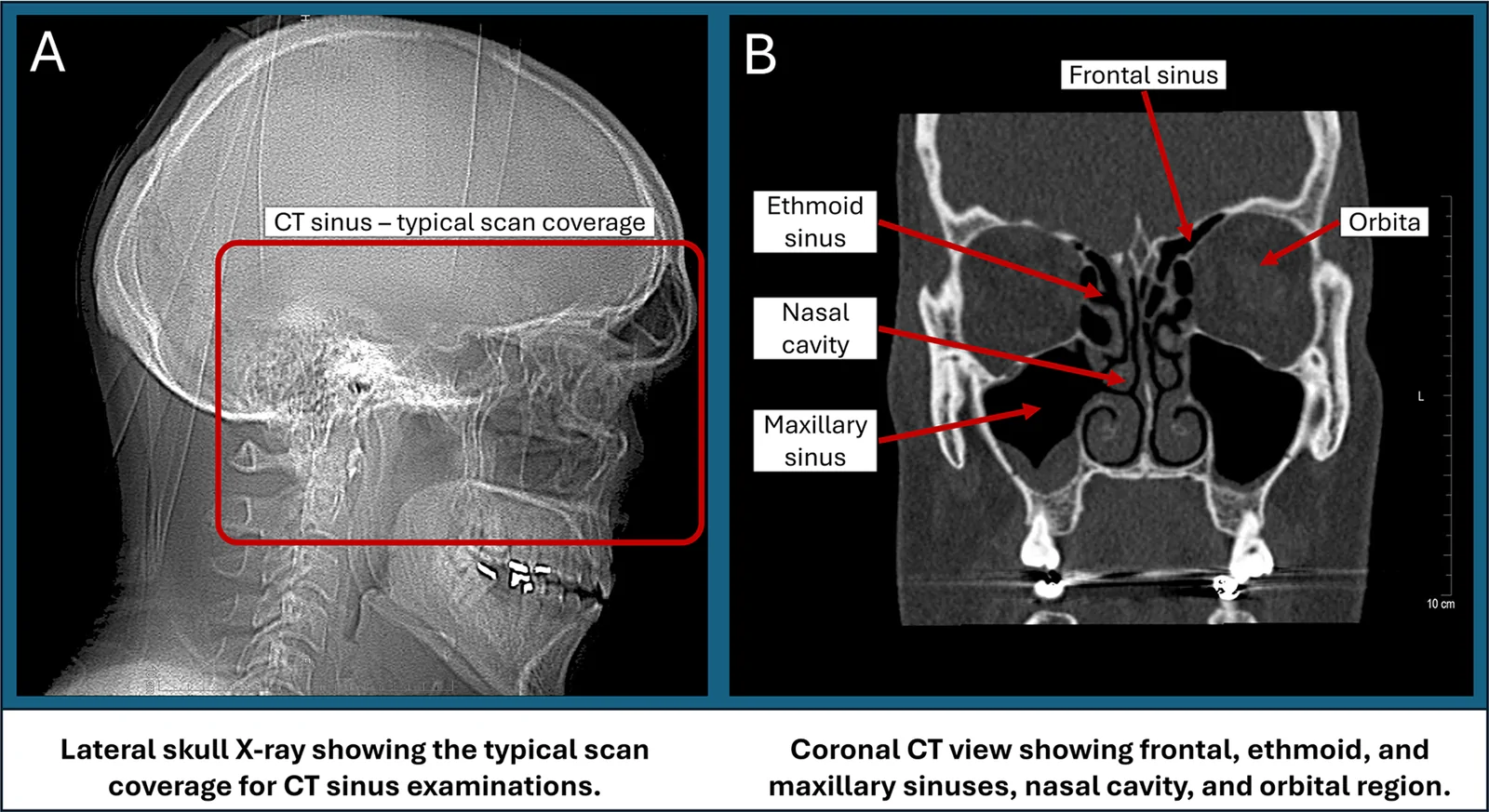
Computed tomography (CT) of the sinuses of a 54-year-old male. **A** is based on the Oslo University Hospital Procedure CT 5E1 Sinuses. 2025. Available from: https://ehandboken.ous-hf.no/document/14178

The purpose of CT sinuses is to visualize the paranasal sinuses (frontal, ethmoidal, sphenoidal, maxillary sinuses), the nasal cavity, and orbit (Fig. 1). The CT scan range extends from the upper dental arch through the paranasal sinuses, including the nasal tip, auricles, and overlying skin. Indications for performing a CT sinus examination in Norway include: chronic sinusitis, suspected fungal sinusitis or malignancies, and preoperative assessment [30, 31]. Acute sinusitis is not among these indications. This aligns with international guidelines and recommendations [32–38]. The European Position Paper on Rhinosinusitis and Nasal Polyps (EPOS) – guidelines delineate acute sinusitis (ARS) as primarily clinical. EPOS states: `Overall CT scan remains the gold standard in the radiologic evaluation of rhinologic disease, notably Chronic rhinosinusitis (CRS). However, in acute rhinosinusitis, the diagnosis is made on clinical grounds, and CT is not recommended unless the condition persists despite treatment, or a complication is suspected` [32, 33, 35–38]. The `Diagnostic tools in Rhinology EAACI position paper` states that CT imaging is generally not advised for diagnosing acute rhinosinusitis, except in cases where complications are suspected or when recurrent rhinosinusitis does not respond to treatment [39]. CT scans of the sinuses are frequently regarded as providing limited medical value for acute sinusitis. Research conducted in the United States and Spain has demonstrated that CT sinus is occasionally performed unnecessarily in cases of acute sinusitis [40, 41]. The research conducted by Sharp et al. demonstrated that, overall, 38% (95% CI, 22%-54%) of patients with acute sinusitis were treated in accordance with the ‘Choosing Wisely’ recommendations regarding CT scans of the sinuses. Clear differences were observed depending on where patients were treated. In the Emergency Departments, 32% (95% CI, 13%-51%) were treated in accordance with the ‘Choosing Wisely’ recommendations, whereas this was the case for 16% (95% CI, 1%-31%) of patients in the Primary Care Clinics [40]. This indicates that, in a large proportion of patients, the ‘Choosing Wisely’ recommendations were not adhered to. Additionally, a pronounced variation was observed, depending on the treatment location of the patients. Consequently, regional disparities in the employment of CT sinus scans may serve as an indicator of low-value care.

In Norway, diagnostic imaging, including CT sinus, is an essential component of specialized healthcare. Both non-profit hospitals and private imaging centres in Norway operate under contractual agreements with healthcare authorities to provide publicly funded imaging services [42]. Regarding outpatient procedures, including diagnostic imaging, co-payment is generally mandated. Access to CT sinus procedures in Norway is not a concern, because availability of the service is limited. But if CT machines and specialist staff (radiologists, radiographers) are involved in low-value CT sinus imaging, the waiting times for emergency cases or clinically indicated oncology and follow-up examinations are consequently prolonged [43].

To the best of our knowledge, there are no registry studies in Norway that specifically examine the utilization of CT sinus at the level of Regional Health Authorities and Health Care Areas for outpatient populations.

## Materials and methods

The aim of this study is to describe the geographical and temporal variation in CT sinus usage among outpatients in Norway, considering factors such as age and sex.

Norway is segmented into four Regional Health Authorities (RHA): Northern Norway RHA, Central Norway RHA, Western Norway RHA, and South-Eastern Norway RHA. These RHAs exhibit considerable variation in population size. *As of 1 January 2023*,* the South-Eastern Norway RHA possesses the largest population*,* amounting to 3*,*120*,*058 inhabitants. The Western Norway RHA has a population of 1*,*138*,*555; the Central Norway RHA has 746*,*835; and the Northern Norway RHA has the smallest population*,* totalling 483*,*536* [44]. The four regional health authorities are responsible for overseeing the hospital trusts (HTs) assigned to them [45]. The HTs are responsible for their respective hospital catchment areas (HCAs). The twenty HCAs had an average population of 261,380, with a standard deviation of 145,709 and a median of 243,100 [44].

In January 2013, Norway’s population was 5,051,275. By January 2023, the population had grown by 8.7% (437,709 individuals), bringing the total to 5,488,984. The gender distribution is relatively balanced across the country. As of January 2023, there were 2,723,514 women and 2,765,470 men in Norway [44].

All imaging procedures in Norway are classified using the Norwegian Classification of Radiological Procedures (NCRP) codes, introduced in 2012 [46]. The NCRP codes define imaging modality, body part, and procedure. CT sinus was identified using the corresponding NRCP code ‘SDX0AD’. CT sinus data for outpatients from 2013 to 2023 were provided by the Norwegian Health Economics Administration (Helfo). Helfo maintains a register of all outpatient examinations conducted in hospitals and imaging centres in Norway, for which costs are covered by the Norwegian Labour and Welfare Administration (NAV) [47]. Included data were: year of examination, age, sex (female/male), and the HCA where the CT sinus was conducted. Age was recorded in 10-year increments (0–9, 10–19, 20–29, 30–39, 40–49, 50–59, 60–69, 70–79, ≥ 80).

The analysis was performed on both unadjusted and adjusted datasets. The rates were standardized for age and sex through direct adjustment to the European standard population, which includes the EFTA – European Free Trade Association, per 10,000 inhabitants [48]. Data regarding population size, age distribution, and sex within the individual RHAs and HCAs were obtained from Statistics Norway [44].

The Mann-Kendall test was employed to statistically evaluate whether the utilization of CT sinus exhibits an increase or decrease over the years. The test statistic associated with the Mann-Kendall test is also known as the score of the Mann-Kendall test. A positive score indicates an upward trend in the use of CT sinus over time, whereas a negative score denotes a descending trend. To identify potential clusters associated with the years evaluated (2013–2023), hierarchical clustering was applied. The objective is to assess whether the utilization of CT sinus in particular segments throughout the entire observation period indicates years during which the pattern of CT sinus usage remains consistently similar. For this purpose, the adjusted CT sinus rates for all HCAs during these years were grouped into clusters with similar values. Only CT sinus rates from consecutive years can be grouped together. Furthermore, longitudinal hierarchical clustering was utilized to assess whether the distinct HCAs could be classified into four clusters aligning with the organizational structure of the four RHAs [49, 50]. This approach aims to provide insights regarding the existence of systematic variations among the four RHAs.

Measures of geographical variation were utilized to compare the spatial differences in CT sinus. To attain a more stable outcome, the average of the years 2013 to 2015 was utilized and compared with 2023, the year after the coronavirus pandemic. The summary of the years 2013 to 2015 is intended to avoid using possible random fluctuations that occur only in a single year as a basis for comparisons. The end of the observation period was not summarised, as restrictions due to COVID-19 still applied in Norway until the year 2022 [51]. The ‘intuitive measures’, namely the coefficient of variation (CV) and the extremal quotient (EQ) were computed, as well as the systematic component of variation (SCV) [52–54]. CV is defined as the standard deviation of the adjusted CT sinus rates of the HCAs divided by the overall mean of the adjusted CT sinus rates across all HCAs [55]. For EQ, the ratio of the highest adjusted CT sinus rate to the lowest adjusted CT sinus rate was utilized [53]. The SCV was calculated using the formula proposed by McPherson et al. [54]. Following Bedane et al., we interpret the SCV as such, that values above 3 indicate differences in clinical practice between HCAs. SCV values ranging from 5 to 10 indicate large variations between HCAs. Values greater than 10 indicate extreme variations between HCAs [56]. Given that EQ and SCV can be affected by outliers, both parameters were further computed utilizing data confined within the 5th and 95th percentiles [57]. These are abbreviated below as EQ_5−95_ and SCV_5−95_.

To evaluate the variable ‘sex’, alongside descriptive statistics, a test for differences in its composition was performed using Mantel-Haenszel standardised statistics [58].

The analyses were conducted at two levels: the Regional Health Authorities and the Hospital catchment areas of the Hospital Trusts. No outpatient CT sinus was reported for Sunnaas HT. Regarding standardisation concerning age and sex, the HCA of Sunnaas HT was integrated into Akershus University Hospital (AHUS) HT due to Sunnaas HT’s geographical proximity to the catchment area of AHUS HT. Our analysis consequently encompasses a total of 19 HTs and includes all HTs in Norway along with their associated HCAs.

All significance tests employed are exploratory in nature, as no hypotheses could be formulated beforehand. An alpha level of 0.05 was utilized as the threshold for significance. For multiple testing procedures, p-values were adjusted in accordance with the Bonferroni correction. Data analysis was conducted using SAS, Stata, and R 4.5.1 [59–61].

The Regional Committees for Medical and Health Research Ethics granted ethical approval for this study (reference 175370).

## Results

### Changes over the years 2013–2023

**Fig. 2:**
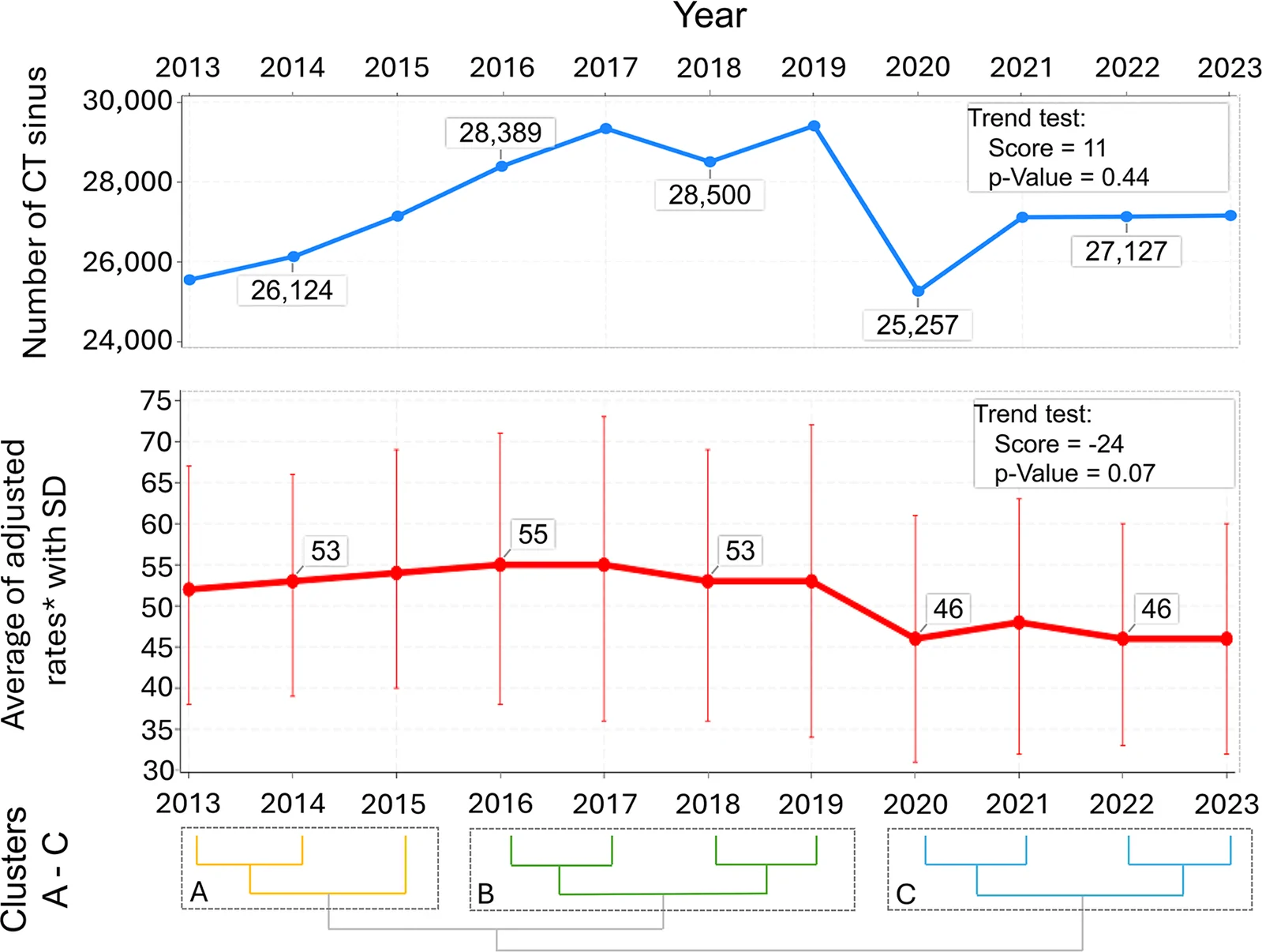
Temporal Trends in CT Sinus Utilization in Norway, 2013–2023. The averages and the standard deviations (SD) are derived from the distribution of adjusted CT sinus rates across the hospital catchment areas (HCAs). A negative score in the trend test indicates a decreasing trend over time, whereas a positive score indicates an increasing trend over time. To examine whether the years of study (2013–2023) formed distinct patterns, hierarchical clustering was applied to the adjusted CT sinus rates across all HCAs. Three primary clusters (**A**, **B**, and **C**) were identified. *Adjusted for sex and age to align with the European Standard Population, including the EFTA – European Free Trade Association. CT, Computed tomography

Between 2013 and 2023, a total of 301,083 CT sinus was performed on outpatients in Norway. When adjusted for sex and age according to the European standard population, this corresponds to an average of 51 (SD ± 16) CT sinus per 10,000 individuals annually. Between 2013 and 2017, the total number of CT sinus increased from 25,643 per year to 29,334, representing a 12.6% rise, and subsequently stabilized at approximately 27,100 CT sinus annually from 2021 onward 2023. The trend test score indicates a positive trend; however, it does not attain statistical significance (*p* = 0.44) (Fig. 2).

The age- and sex-adjusted rates per 10,000 individuals exhibit an upward trend until 2016/2017, after which they level off, followed by a decline culminating in 2022 with 46 CT sinus per year. The trajectory of the adjusted rates over the period from 2013 to 2023 generally commensurates with that of the unadjusted number of CT sinus. In contrast to the absolute numbers, the adjusted rates at the end of the observation period are lower than those observed in 2013 and 2014. The trend test score is negative, with a p-value of 0.07 (Fig. 2).

**Fig. 3:**
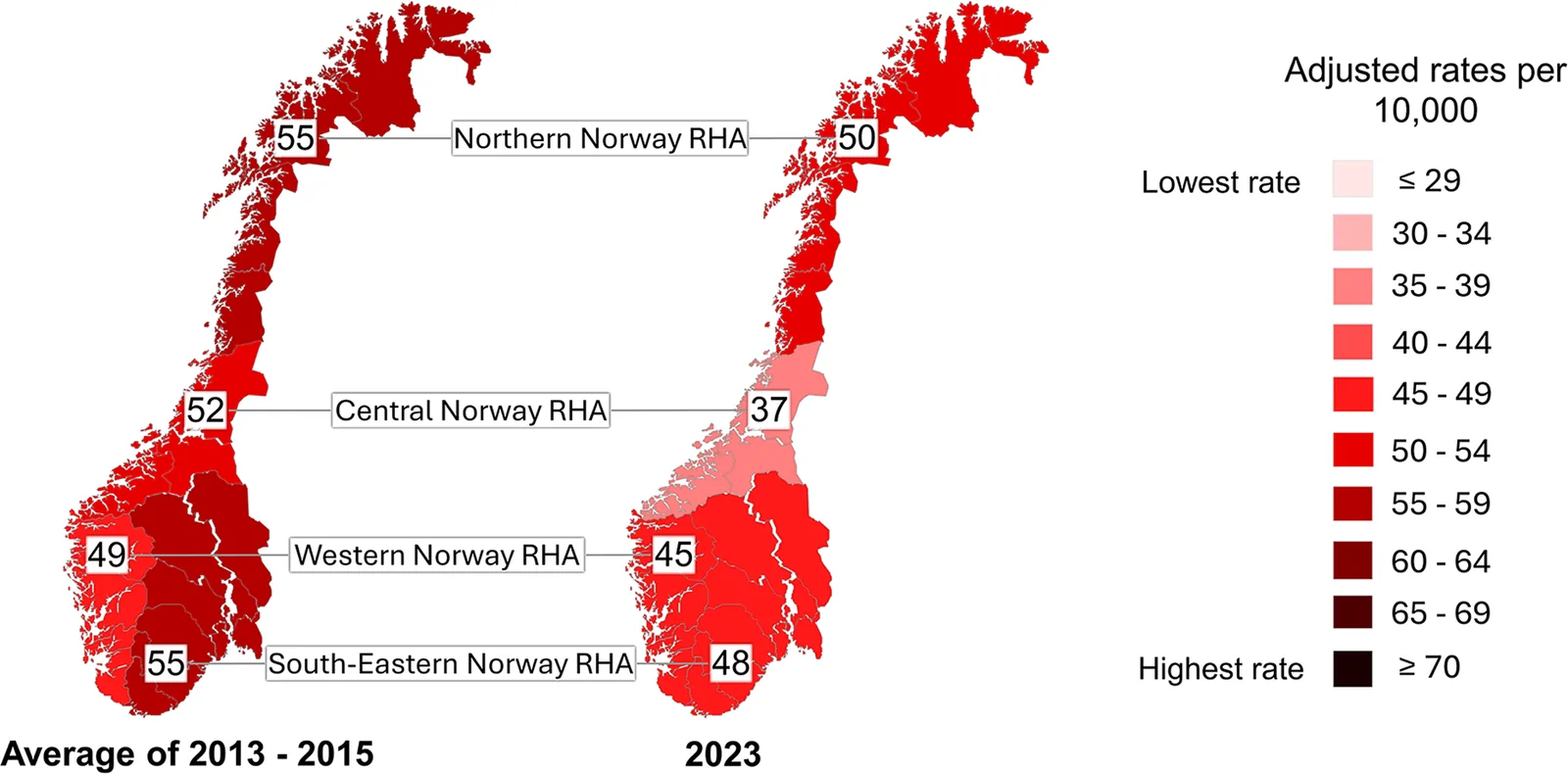
Adjusted computed tomography sinus rates by regional health authority (RHA): Comparison of 2013–2015 and 2023. *Adjusted for sex and age to align with the European Standard Population, including the EFTA – European Free Trade Association. RHA, Regional Health Authority

An analysis of data from the four Regional Health Authorities indicates that, when comparing averages from 2013 to 2015 with those in 2023, adjusted CT sinus rates per 10,000 declined (Fig. 3).

**Fig. 4:**
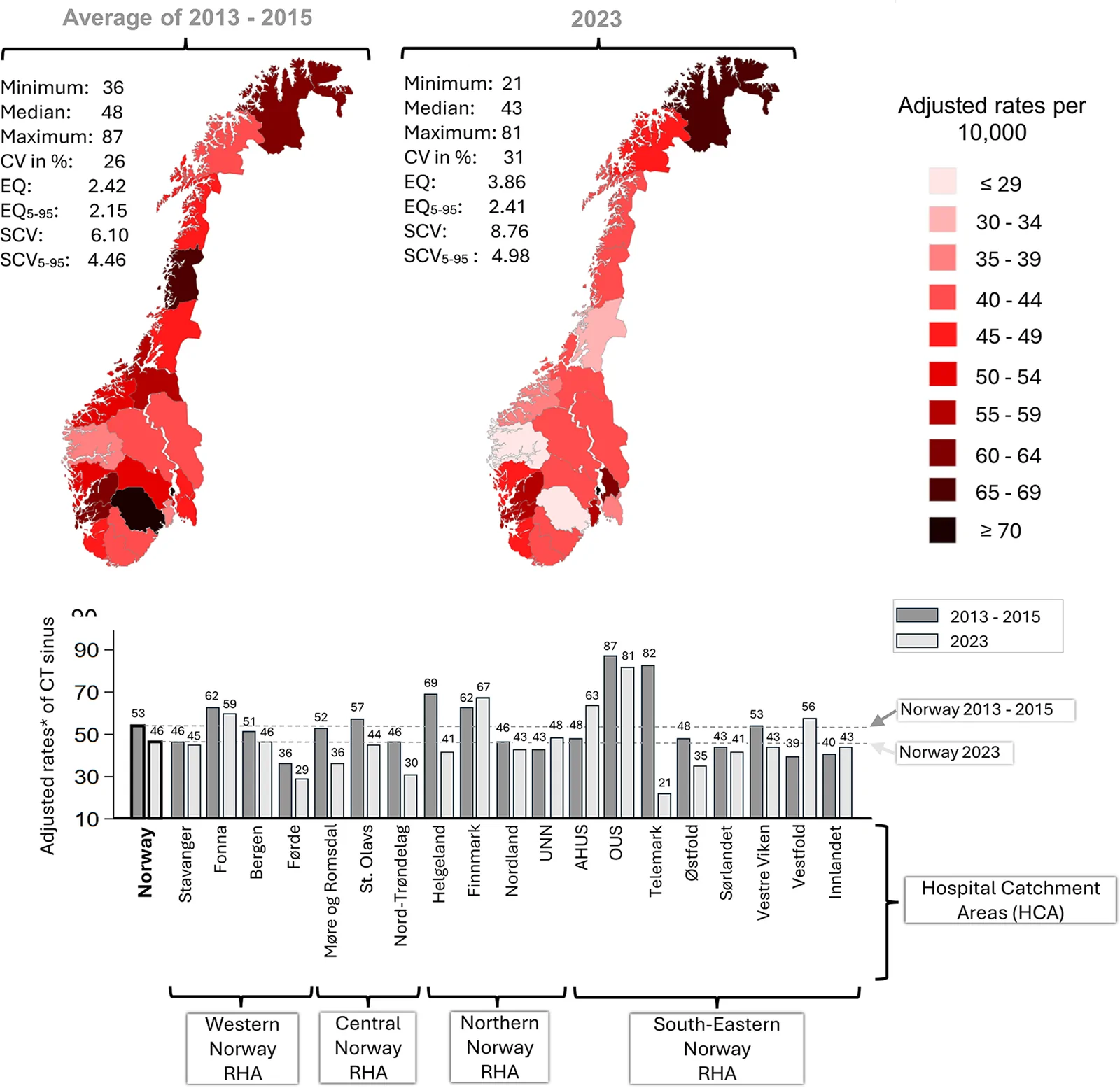
Adjusted rates of CT sinus by hospital catchment areas (HCA) for the years 2013–2015 and for 2023. *Adjusted for sex and age to align with the European Standard Population, including the EFTA – European Free Trade Association. CT, Computed tomography; CV, coefficient of variation; EQ, extremal quotient; SCV, systematic component of variation (McPherson formula); AHUS, Akershus University Hospital; OUS, Oslo University Hospital; UNN, University Hospital of North-Norway

The trend test scores are negative across all four RHAs, indicating a decreasing trend over time for each (Table 1). This negative trend is also evident in most HCAs. Nevertheless, five HCAs demonstrated an increase in CT sinus rates when comparing the periods of 2013–2015 with 2023. These include AHUS (48 vs. 63), Finnmark (62 vs. 67), Innlandet (40 vs. 43), University Hospital of North-Norway (UNN) (43 vs. 48), and Vestfold (39 vs. 56) (Fig. 4).

**Table 1:** Adjusted CT sinus rates for the four regional health authorities

Year	Western Norway RHA	Central Norway RHA	Northern Norway RHA	South-Eastern Norway RHA
2013	49	50	53	55
2014	48	52	55	54
2015	50	53	57	56
2016	49	52	57	57
2017	48	52	56	58*
2018	48	50	51	57
2019	51	46	51	58*
2020	44	41	48	48
2021	45	42	50	50
2022	42	40	49	50
2023	45	37*	50	48
Score of the Trend Test	-26 *p* = 0.19	-39 *p* = 0.011	-30 *p* = 0.09	-13 *p* = 0.99

All rates are adjusted for sex and age to align with the European Standard Population, including European Free Trade Association (EFTA). The minimum and maximum values are denoted with an asterisk (*). A negative score on the trend test indicates a decreasing trend over time. The corresponding p-values have been corrected for multiple testing according to the Bonferroni Adjustment. CT, Computed tomography; RHA, Regional Health Authorities

### Geographical variations

The median adjusted rate of CT sinus for the RHAs is 50, with rates spanning from 37 to 58 per 10,000. In 2023, the Central Norway RHA’s adjusted CT sinus rate is notably lower (37 per 10,000) compared to the rates of the other three RHAs, which range from 45 to 50 per 10,000 (Fig. 3). The adjusted rates of the HCAs exhibit notable variabilities (Fig. 4). The median number of adjusted CT sinus performed annually in HCAs from 2013 to 2023 is 47 scans per 10,000 inhabitants, with a minimum of 32 and a maximum of 96.

Among the HCAs, Oslo University Hospital (OUS), Telemark, and Helgeland have the highest adjusted rates per 10,000 for the period 2013–2015 (87, 82, and 69). In 2023, HCA OUS continued to hold the highest adjusted rate at 81, followed by HCA Finnmark at 67, and HCA AHUS at 63 per 10,000. The lowest adjusted rates per 10,000 in 2023 are observed in the HCAs of Telemark [21], Førde [29], and Nord-Trøndelag [30]. However, the RHAs demonstrate a narrower range of adjusted CT sinus rates than the HCAs (Figs. 3 and 4).

The measures of variation indicate an increase in geographical variability when comparing the averages for 2013–2015 with those for 2023. The CV rises from 26% to 31%, the EQ_5−95_ increases from 2.15 to 2.41, and the SCV_5−95_ from 4.46 to 4.98. Values for the SCV_5−95_ exceeding 3 suggest differences in medical practice. Additionally, the SCV values increased from 6.10 during 2013–2015 to 8.76 in 2023, indicating a high level of geographical variation (Fig. 4).

**Fig. 5:**
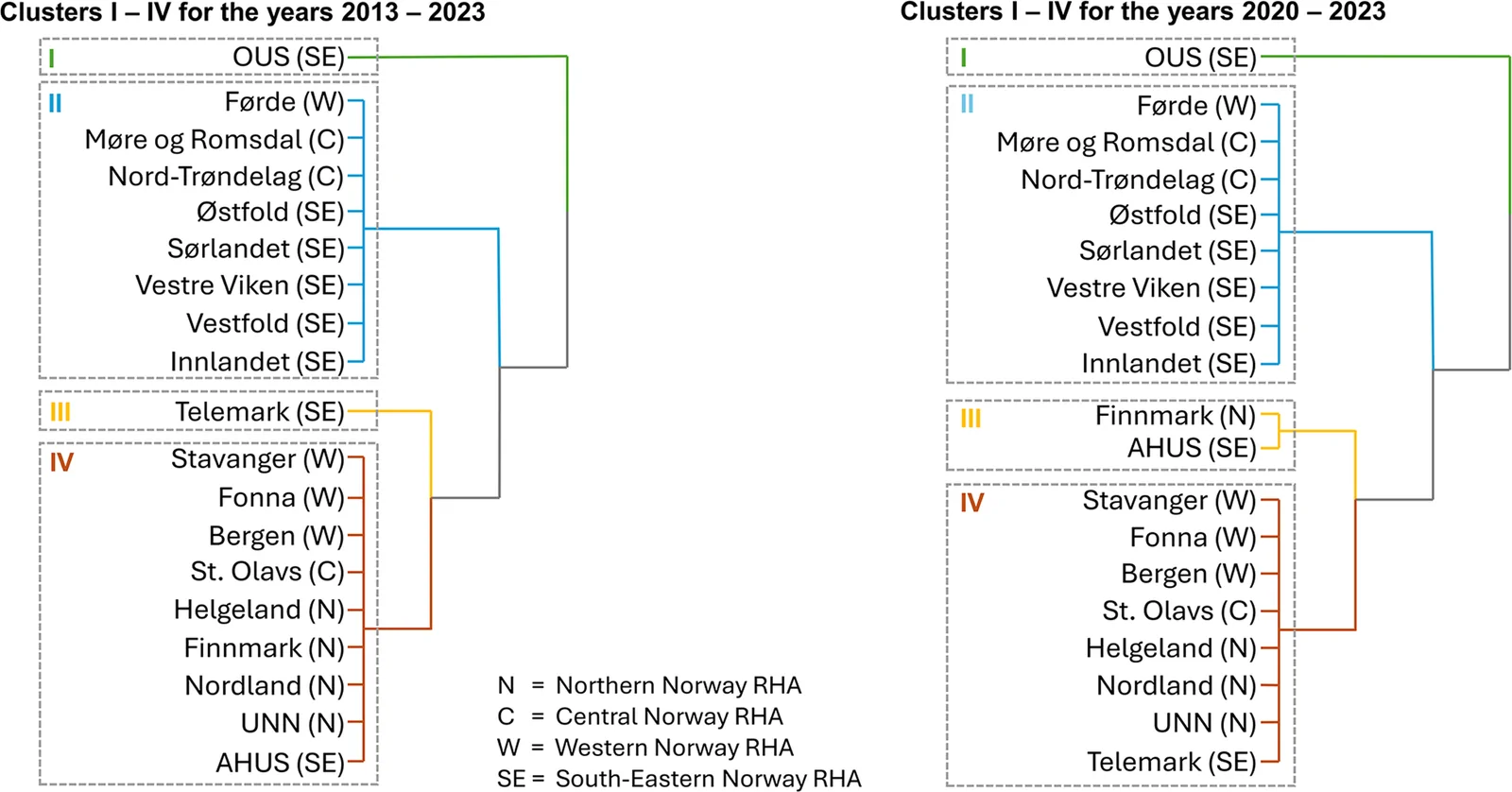
Longitudinal hierarchical clustering of hospital catchment areas: Entire period (2013–2023) and years (2020–2023). The two dendrograms illustrate the results of the longitudinal hierarchical clustering analysis performed on the adjusted CT sinus rates* for all hospital catchment areas (HCAs). The aim was to determine whether the HCAs can be classified into four distinct clusters that correspond to the organisational structure of the four Regional Health Authorities (RHAs). *Adjusted for sex and age to align with the European Standard Population, including the EFTA – European Free Trade Association. AHUS, Akershus University Hospital; OUS, Oslo University Hospital; UNN, University Hospital of North-Norway

The outcomes of the longitudinal hierarchical cluster analysis indicate the presence of two minor clusters, each consisting of one or two HCAs, as well as two larger clusters comprising eight or nine HCAs. These four clusters, as identified through the analysis, do not align with the four RHAs either for the periods 2013–2023 or for the more truncated interval of 2020–2023. HCA OUS forms a separate, distinct cluster and contrasts with the remainder of the country. The observation that the identified clusters do not correspond with the organisational structure of the RHAs indicates that there are probably no systematic disparities in the application of CT sinus across the RHAs. Consequently, the variations observed among the HCAs cannot be attributed to processes and structures specific to a particular RHA (Fig. 5).

### Age and sex

**Fig. 6:**
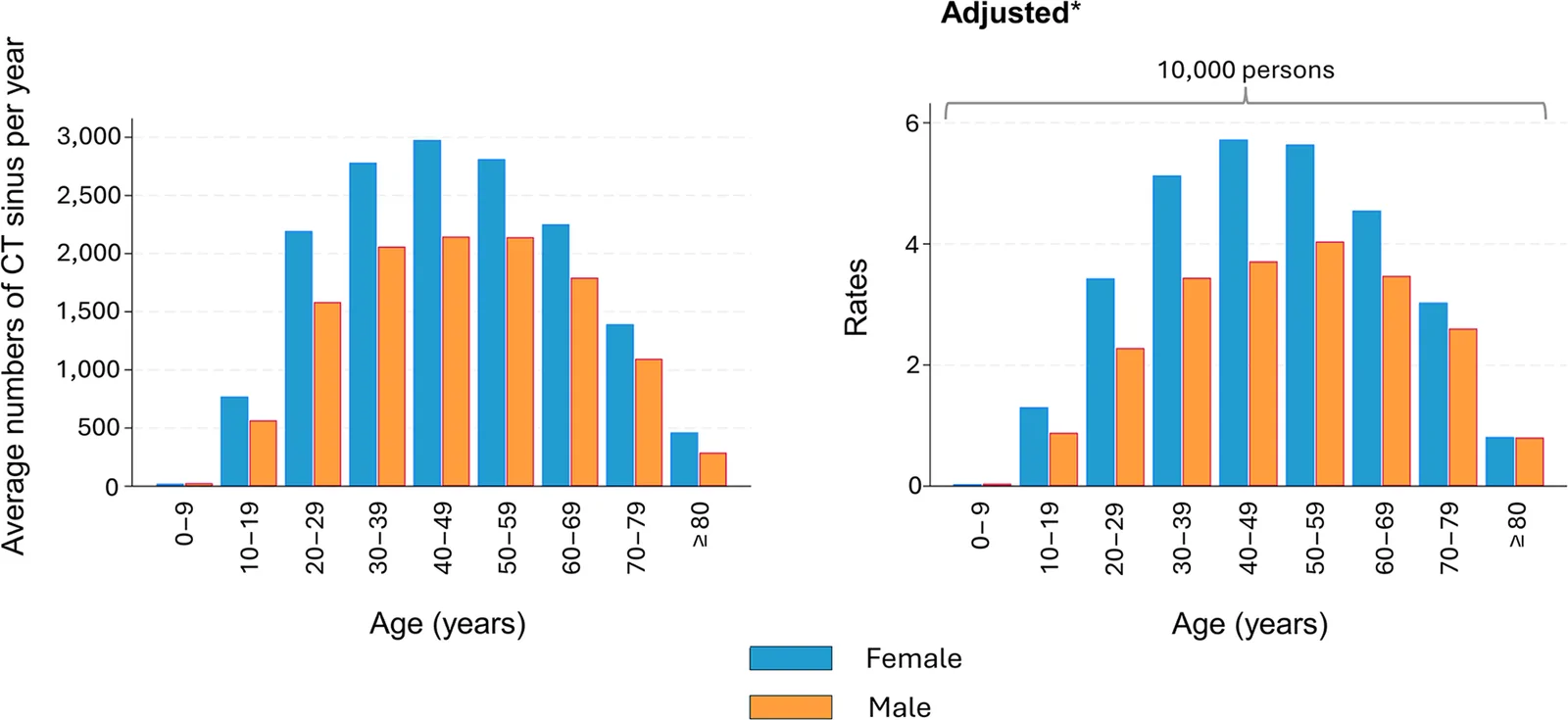
Average CT Sinus Utilization by Age Group and Sex, 2013–2023. *Adjusted for sex and age to align with the European Standard Population, including the EFTA – European Free Trade Association. CT, Computed tomography

The age distribution of individuals undergoing CT sinus follows approximately a normal distribution. There are only a few CT scans performed in the younger and older age groups (see Fig. 6). In the 0–9 age cohort, an average of 47 CT sinus were conducted annually across Norway. In 2022, the total number of CT sinus within this age group nationwide was 40, and in 2023, only 9. By comparison, the average for the years 2013–2015 was 65 CT sinus per year in the 0–9 age group. For individuals aged 80 years or older, the unadjusted total number of CT sinus per annum remains relatively constant. During 2013–2015, the average in this age group was 743; in 2022, it was 704, and in 2023, 698 CT sinus.

**Fig. 7:**
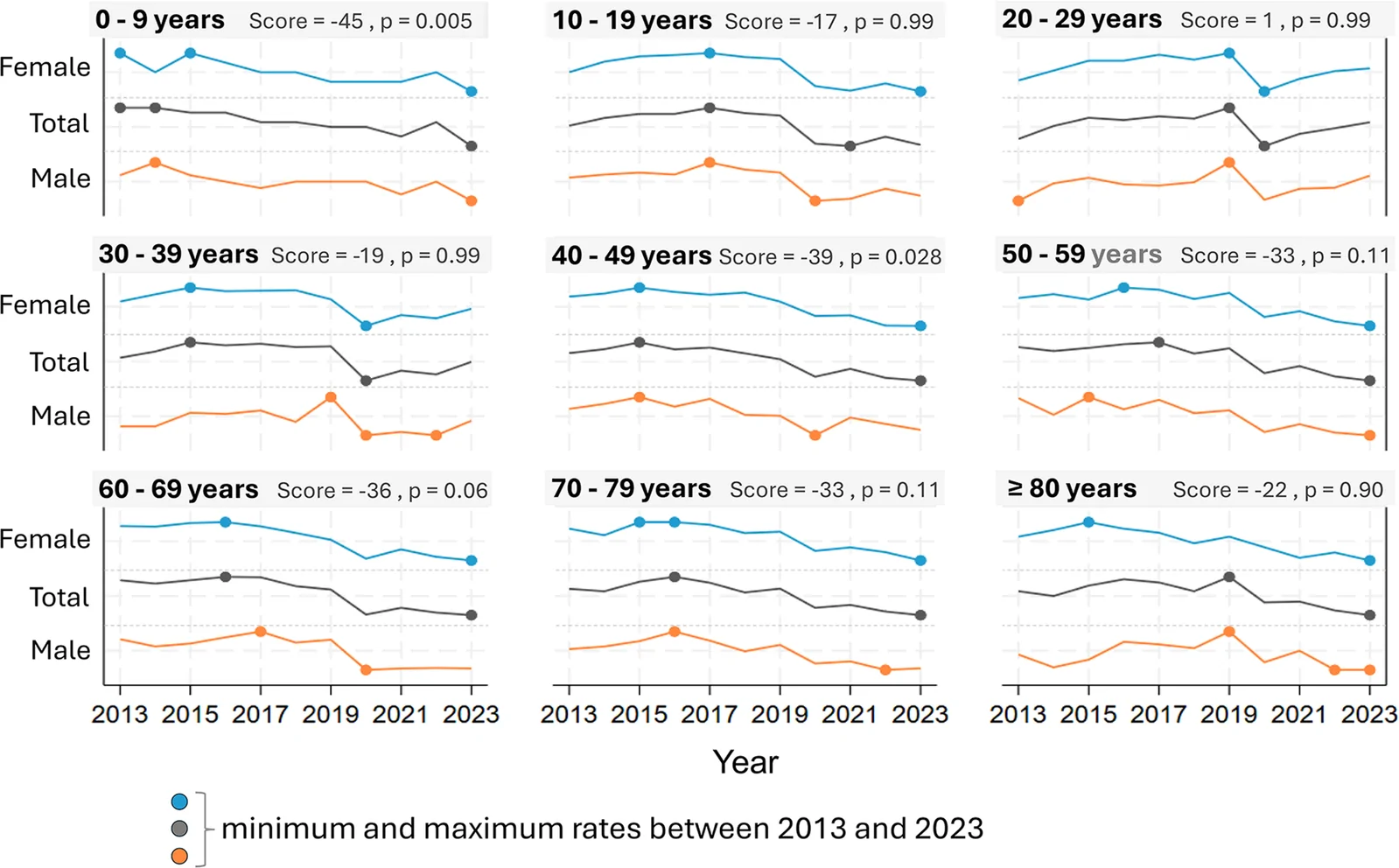
Adjusted CT sinus rates over time for females, males, and combined, by age group. The sparkline plot illustrates the trend of the adjusted CT sinus rates separately for females, males, and the combined data for both men and women (Total). The coloured dots denote the minimum and maximum values observed between 2013 and 2023. The Score represents the trend test statistic for all groups combined (females and males). A negative trend test score indicates a decreasing trend over time, whereas a positive score indicates an increasing trend over time. The associated p-values are corrected for multiple testing according to the Bonferroni correction. *Adjusted for sex and age to meet the European Standard Population (including EFTA – European Free Trade Association). CT, Computed tomography

The adjusted rates show a negative trend test score across all age groups, indicating a decreasing trend over time in nearly all categories, except for individuals aged 20 to 29 (Fig. 7). Notably, since 2022, the adjusted CT sinus rates have increased in the 20 to 29 age group. Additionally, for the 30 to 39-year group, rates are increasing, but only in 2023. The upward trend in adjusted rates for these two age groups affects both males and females (see Fig. 7). In absolute numbers, 3,482 CT sinus were performed nationwide in Norway in 2020 within the 20–29 age group. By 2023, this number increased to 3,991, representing a 14.6% rise.

CT sinus is conducted with greater frequency among female patients (Fig. 6). This pattern has been consistently observed annually from 2013 to 2023 across all RHAs. It pertains to both standardised rates and absolute numbers. The female-to-male ratio for undergoing CT sinus is 1.42 (SD ± 0.11), with a median value of 1.41 for the age-, sex-, and population-adjusted rates over the specified period and across all Regional Health Authorities (Table 2). In the year 2023, the observed difference in Mantel-Haenszel standardised estimates between females and males is 0.00131 (95% CI: 0.001041–0.001573), a difference that is statistically significant (*p* < 0.0001). Over the period from 2013 to 2023, the cumulative difference in Mantel-Haenszel standardised estimates amounts to 0.00154 (95% CI: 0.001504–0.001576), which is also statistically significant (*p* < 0.0001). The trend test scores from 2013 to 2023 regarding the female-to-male ratio indicate a decline in the female-to-male ratio within the Northern Norway RHA and the South-Eastern RHA. Conversely, the Western Norway RHA maintains a relatively consistent female-to-male ratio throughout this period (see Table 2).

**Table 2:** Average female-to-male ratio of CT sinus across regional health authorities

Year	Western Norway RHA	Central Norway RHA	Northern Norway RHA	South-Eastern Norway RHA
2013	1.37	1.45	1.54	1.32
2014	1.29	1.38	1.84*	1.35
2015	1.41	1.42	1.58	1.35
2016	1.38	1.48	1.51	1.41
2017	1.40	1.46	1.53	1.32
2018	1.44	1.53	1.59	1.33
2019	1.37	1.45	1.49	1.30
2020	1.32	1.46	1.50	1.31
2021	1.40	1.47	1.49	1.26
2022	1.29	1.44	1.51	1.29
2023	1.37	1.38	1.45	1.25*
Score Trend Test	-6 *p* = 0.69	0 *p* = 0.99	-31 *p* = 0.019	-35 *p* = 0.008

The table presents the average female-to-male ratios for each Regional Health Authority per year, derived from data adjusted for sex and age to conform to the European Standard Population, including the European Free Trade Association (EFTA). A negative score on the trend test indicates a decreasing trend over time. The minimum and maximum values are indicated with an asterisk (*). RHA, Regional Health Authorities

## Discussion

The adjusted rates and absolute numbers of CT sinus declined during the evaluation period. This trend was observed in both the four RHAs and the majority of HCAs (14 out of 19). This may be linked to the general trends of imaging in Norway and to the ‘Choosing Wisely’ campaign, which seeks to reduce the use of low-value care. In Norway, brain Magnetic Resonance Imaging (MRI) and head CT for uncomplicated headaches are incorporated into the ‘Choosing Wisely’ campaign of the Norwegian Radiological Society [62]. Additionally, CT sinus in patients with uncomplicated acute rhinosinusitis is included in the ‘Choosing Wisely’ campaign of the Norwegian Society of Otorhinolaryngology, Head and Neck Surgery [63]. The campaign promotes avoiding CT sinus in instances of uncomplicated acute rhinosinusitis. It may have contributed to a reduction in other low-value radiological tests and contributed to the decline of CT sinus rates over the years. It is worth noting that in approximately the same period (2013 to 2022), the overall number of head MRI examinations increased by 16% [64].

The most notable change in CT sinus rates from one year to the next occurred with the start of the coronavirus pandemic, particularly when comparing 2019 with 2020. After the pandemic began, the adjusted average rates did not increase again (see Fig. 2). This phenomenon may be attributed to decreased utilization following the pandemic. However, given that there are no capacity limitations, there might be another reason: it is more likely that this trend indicates a shift toward more appropriate care, specifically a reduction in low-value imaging. The largest decrease in adjusted CT sinus rates between 2013 and 2015 and 2023 was observed in the HCA Telemark. Between 2013 and 2015, the average was 82, ranking among the highest within the HCAs, and in 2023, it decreased to 21 per 10,000. Since 2015, a consistent decrease in the frequency of CT sinus across all age groups has been documented at HCA Telemark. This trend may have been further reinforced by the ‘Choosing Wisely’ Campaign [63].

Both the adjusted CT sinus rates and the measures of variation indicate geographical differences. These variations have not diminished when comparing data from 2023 with that from 2013 to 2015; rather, they have increased (see Fig. 4). The increase in the CV can be explained as follows: The mean of the adjusted CT sinus rates among the HCAs decreased from 53.2 during 2013–2015 to 45.9 in 2023. Conversely, the variability of the adjusted CT sinus rates, as measured by the standard deviation, experienced a slight increase from 13.5 in 2013–2015 to 14.0 in 2023. Since the CV is calculated as the ratio of the standard deviation to the overall mean, this results in an elevation of the coefficient of variation. Prior studies conducted in Norway have also identified geographical differences in outpatient imaging [17, 65, 66].

In the present study, data were analysed according to the Regional Health Authorities (RHAs) and the Catchment Areas (HCAs) associated with the Hospital Trusts (HTs). The evaluation did not consider the residence of individuals who underwent a CT sinus. Patients undergoing examinations at prominent centres, such as those in HCA OUS, may reside in regions of Norway that belong to different HCAs rather than HCA OUS. This partially accounts for the higher frequency of CT sinus performed at HCA OUS compared to other HCAs. In 2023, after adjustment for sex and age, there were 81 CT sinus at HCA OUS per 10,000 individuals, compared to the national average of 46. HCA OUS, which houses two major national medical centres - Ullevål Hospital and Rikshospitalet – as well as the Radiumhospitalet, all of which are part of OUS, serves patients nationwide. Moreover, its unique status is further illustrated by the fact that HCA OUS constitutes its own distinct cluster (see Fig. 3).

Based on our analysis, the geographical variations are not attributable to the RHAs, as no HCA clusters have been identified that fully or substantially correspond with the RHAs. According to Wennberg’s argumentation, it appears most probable that these variations stem from specific characteristics at the HCA level or from individual medical facilities, teams, or routines [67].

The increase in CT sinus among individuals aged 20 to 29 from 2020 onwards is also associated with a rise in the absolute number of CT sinus. Between 2020 and 2023, there was an absolute increase of 509 cases (14.6%), despite a 1.1% population decline in this age group from January 1, 2020, to January 1, 2024 [44]. We lack access to data regarding outpatient physician consultations for individuals aged 20–29. Nonetheless, it was observed that within the 20–39 age cohort, outpatient physician consultations that potentially resulted in referrals for CT sinus increased by a modest 1.3% overall between the years 2020 and 2023 (Supplement Table 1). The conditions headache and migraine are not included in this, as data categorised for these two conditions are not available [44]. Nevertheless, we suspect that for this age group (20–29), more referrals for CT sinus will be issued per capita in 2023 than in 2020, as shown in Fig. 7.

Our findings indicate that, irrespective of geographical factors, the number of CT sinus conducted on women significantly exceeds that on men. We were unable to find literature that also reports these observations. Data from Statistics Norway for the year 2023 demonstrate that women aged 20–59 are considerably more likely than men to seek outpatient consultations for acute respiratory infections and diseases affecting the mouth, salivary glands, and jaws. The ratio of women to men, based on adjusted consultation rates for these two conditions, ranges from 1.22 to 1.63. Similar ratios are observed in the unadjusted data (see Supplementary Table 1). International studies further corroborate that sinusitis is diagnosed more frequently in women than in men, indicating a consistent trend. The Summary Health Statistics for U.S. Adults report age-adjusted prevalence percentages for sinusitis of 9% (SD ± 0.30) for men and 14.5% (SD ± 0.34) for women [68]. A Canadian study published in 2003 reports a prevalence of rhinosinusitis in persons aged 12 years and older of 3.4% (95% CI: 3.0–3.9%) for male versus 5.7% (95% CI: 5.2–6.1%) for women [69]. The higher prevalence of rhinosinusitis may partly explain why female are more often referred to CT sinus. However, for the most common reason for consultation, ‘other upper respiratory tract conditions’, the same difference between women and men is not observed in Norway within the 20–39 and 40–59 age groups. The female/male ratio here is 1.11 and 0.97 for adjusted rates, respectively (Supplement Table 1). This indicates that other factors lead to women undergoing CT sinus more frequently. Conversely, data regarding consultations due to headaches or migraines are unavailable; nevertheless, numerous other factors may contribute to the higher frequency of CT sinus in women. Ference et al. note that migraine headaches, which are more common in women, are often misdiagnosed as rhinosinusitis, and that women report more severe symptoms for the same severity of rhinosinusitis [70]. CT sinus may constitute low-value care [62, 71]. Therefore, it is advisable, particularly with female patients, to exercise caution when requesting CT sinus - such as in cases of acute sinusitis - even when rhinosinusitis substantially impairs quality of life, more so than in males.

Considering the generally high level of imaging in Norway [15, 72], the overall decrease in the number of examinations may indicate an increased attention to appropriate care. However, the increased geographical variations are of clinical importance and raises concerns for health policy within health systems. This is particularly pertinent to healthcare systems with universal coverage, where equity is a fundamental principle.

### Strengths and limitations

Our analysis was exclusively based on CT sinus data retrieved from outpatient populations. Inpatients were deliberately excluded, as we only possess definitive information that CT sinus were conducted on inpatients at 28% of HCAs. Moreover, we lack data pertaining to inpatients for the subsequent year following the COVID-19 pandemic, specifically for the year 2023. The median ratio of outpatient-to-inpatient CT sinus in an available dataset is 12:1, with an interquartile range of 7:1 to 22:1. Given the substantial variability within the data and the inability to obtain additional retrospective data from preceding years, we limited our analysis to outpatient data, where we have a near complete data set. This study does not include imaging paid for privately or through voluntary health insurance. Voluntary health insurance has a minimal impact, representing less than 1% of total health expenditure in Norway in 2017 [42]. However, the number of CT sinus financed through such means remains unknown. Consequently, our data likely underestimates the actual figure, although the precise extent of this underestimation is unknown. There were only missing values for two variables throughout the entire dataset. For the variable ‘age-group’, a total of 167 missing values were recorded, representing 0.055%. The variable ‘sex’ had a total of 7 missing values, accounting for 0.002%. Given these minimal percentages, we opted not to impute the missing data.

To achieve more reliable results when assessing temporal variations, the years 2013–2015 were aggregated for certain analyses. This aggregation is consistent with the fact that these years constitute a single cluster, Cluster A, as demonstrated by the hierarchical clustering analysis (see Fig. 2). Based on the hierarchical cluster analysis, we could also have combined the years 2020–2023 or 2022–2023. However, for the comparison period at the end of the observation period, we only used the year 2023 and did not combine it with 2022 to avoid distortions caused by effects related to the coronavirus pandemic. At the start of the year 2022, not all restrictions related to the pandemic had been lifted in Norway. On 12 February 2022, the Norwegian Government formally repealed all regulatory measures pertaining to COVID‑19 [51]. As a result, statements regarding the measures of variation may be subject to a certain degree of uncertainty.

We chose to make the adjustment using the European Standard Population (including EFTA – Europe) because this standard population is very similar to the Norwegian population structure and allows for easy comparison with other European countries.

The data accessible to us does not permit the identification of the reasons for consultation that resulted in a referral for CT scans of the sinus. Therefore, it is conceivable that CT scans are conducted in cases of acute sinusitis, for instance, as has been documented in the United States [40]. In Norway, an investigation should be conducted to determine the specific diagnoses for which CT sinus are performed, thereby enabling the implementation of measures to reduce unnecessary CT sinus if deemed necessary.

Temporal and geographical variations do not offer conclusive insights into the appropriate level of care and, consequently, cannot quantify underuse or overuse. Such variations denote the prevalence of low-value services and may suggest that the quality of care is influenced by the healthcare supply available in specific regions [67].

## Conclusions

In Norway, there has been a notable decline in the rates of CT sinus over time. This trend is partially attributable to modifications resulting from the coronavirus pandemic, but it is also presumably influenced by the ‘Choosing Wisely’ campaign. Concurrently, geographical variability, which was already evident between 2013 and 2015, has tended to increase until 2023. This may indicate that patients are receiving different treatment approaches in the various HCAs. Given that CT sinus are potentially low-value investigations, it is plausible that patients in certain hospital catchment areas in Norway are undergoing unnecessary procedures. Therefore, when referring patients to a CT scan of the sinuses, it is essential to verify that there is a legitimate indication for conducting such a procedure. Acute sinusitis is explicitly excluded from this consideration. Of particular importance is the observation that the frequency of CT sinus conducted on women significantly exceeds that on men. Many of these examinations performed on women could also represent low-value care.

## Electronic Supplementary Material

Below is the link to the electronic supplementary material.

## Data Availability

The datasets generated and analysed during the current study are not publicly available due to regulation by the REC (Research Ethics). However, aggregated data are available from the corresponding author on reasonable request.

## Abbreviations

AHUS: Akershus University Hospital
ARS: Acute sinusitis
CRS: Chronic rhinosinusitis
CT: Computed tomography
CV: Coefficient of variation
EFTA: European Free Trade Association
EPOS: European position paper on rhinosinusitis and nasal polyps
EQ: Extremal quotient
HCA: Hospital catchment area
Helfo: Norwegian Health Economics Administration
HT: Hospital trust
NAV: Norwegian Labour and Welfare Administration
NCRP: Norwegian classification of radiological procedures
OECD: Organisation for Economic Co-operation and Development
OUS: Oslo University Hospital
RHA: Regional health authority
SCV: Systematic component of variation
UNN: University Hospital of North-Norway
