# Supplementary material for: CT sinus imaging utilization in Norway: trends over time and variation by region, age, and sex
Source: Res Health Serv Reg. 2026 Sep 2;5:13. doi: 10.1007/s43999-026-00094-4 (PMC13538304; doi:10.1007/s43999-026-00094-4)
Supplement: Supplementary file 1 [file 43999_2026_94_MOESM1_ESM.pdf]

Additional file 1

Supplement Table 1 - Statistics on all outpatient consultations in Norway in 2023 that may lead to a CT sinus referral.

	**Age Group 20-39**			**Age Group 40-59**		
**Condition**	**Female**<br>n (%)<br>adj (%)	**Male**<br>n (%)<br>adj (%)	**Ratio female/ male**<br>unadjusted<br>adjusted	**Female**<br>n (%)<br>adj (%)	**Male**<br>n (%)<br>adj (%)	**Ratio female/ male**<br>unadjusted<br>adjusted
Acute respiratory infections	943 (59)<br>13 (60)	649 (41)<br>8.6 (40)	1.45<br>1.52	508 (54)<br>7.1 (55)	436 (46)<br>5.9 (45)	1.17<br>1.22
Other upper respiratory tract conditions	11,510 (51)<br>161 (53)	10,869 (49)<br>145 (47)	1.06<br>1.11	7,786 (48)<br>110 (49)	8,356 (52)<br>113 (51)	0.93<br>0.97
Other respiratory diseases	448 (45)<br>6 (46)	553 (55)<br>7.4 (54)	0.81<br>0.85	1,535 (50)<br>22 (51)	1,535 (50)<br>21 (49)	1.00<br>1.04
Diseases of mouth, salivary glands and jaws	4,204 (61)<br>59 (62)	2,715 (39)<br>36 (38)	1.55<br>1.63	3,455 (57)<br>49 (58)	2,583 (43)<br>35 (42)	1.34<br>1.40
Fractures of skull, facial bones and neck region	526 (26)<br>7.3 (27)	1,489 (74)<br>20 (73)	0.35<br>0.37	466 (36)<br>6.6 (37)	821 (64)<br>11 (63)	0.57<br>0.59
Concussion and other internal head injuries without skull fracture	1,673 (49)<br>23 (50)	1,743 (51)<br>23 (50)	0.96<br>1.01	1,304 (50)<br>18 (51)	1,311 (50)<br>18 (49)	0.99<br>1.04
**Total**	19,304 (52)<br>270 (53)	18,018 (48)<br>240 (47)	1.07<br>1.12	15,054 (50)<br>212 (51)	15,042 (50)<br>203 (49)	1.00<br>1.04

The table shows the number of consultations for each condition, including total counts and adjusted rates per 10,000 individuals, stratified by sex and age. The percentages represent the proportion of men or women within each condition and age group. The female-to-male ratio is derived from the adjusted values, which are adjusted for sex and age to align with the European Standard Population, including the European Free Trade Association (EFTA). Conditions related to headache and migraine are not included due to the absence of data for these two conditions. Note: 'adj' denotes adjustment for sex and age per 10,000.
